# The Effect of Short-Term Aerobic Exercise on Depression and Body Image in Iranian Women

**DOI:** 10.1155/2013/132684

**Published:** 2013-11-21

**Authors:** Sareh Zarshenas, Parsa Houshvar, Ali Tahmasebi

**Affiliations:** Department of Occupational Therapy, School of Rehabilitation Sciences, Isfahan University of Medical Sciences, Isfahan, Iran

## Abstract

The purpose of this study was to determine the effect of short-term aerobic exercise on depression symptoms and body image attitudes among Iranian women. In this quasiexperimental study, 82 females were assigned to experimental group (aerobic exercise group, *n* = 41) or control group (waiting list, *n* = 41) and evaluated by Beck Depression Inventory-second edition (BDI-II) and Multidimensional Body Self-Relation Questionnaire (MBSRQ), respectively. The experimental group received four-week aerobic exercise program, and control group had been asked to wait for the next four weeks. Results of this study confirmed the significant decrease in depression symptoms at the experimental group compared to control group (*P* < 0.5). For the body image dependent variables, significant improvement was also found in appearance evaluation, appearance orientation, health orientation, and illness orientation in aerobic exercise group (*P* < 0.5).

## 1. Introduction

Negative body image and body-image dissatisfaction are two cognitive symptoms in depression which can also be the cause of depression [1–4]. Body image refers to the accuracy of an individual's perception about body size and thoughts and feelings associated with his or her body [5]. It has profound implications for various psychological disorders and disturbances such as low self-esteem, depression, social anxiety, sexual dysfunction, anorexia nervosa, and body-dysmorphic disorder [2, 6].

Over the last decades, increasing sociocultural emphasis on physical attractiveness and fitness by most of the mass media has raised very deep concern among psychologists and sociologists which may contributed to the increasing rate of body-image dissatisfaction among women [2, 7–9].

Traditional treatment of these disorders is based on pharmacology and psychotherapy, which can be very expensive and time consuming [10]. The symptoms of depression do not however respond to antidepressant drugs until they have been administered for 10 to 20 days which also suggests that a similar period of exercise is needed for adaptations in neural regulation. Thus, approximately four-week adherence to aerobic exercise may be needed to observe changes in mood state and depression. In aerobic exercise sessions that were conducted in gymnasium settings, motivation to get exercise is provided, respectively. However, it remains to be seen how effective short-term exercise could be. In addition, recent research has not considered the effect of short-term exercise on body image and depression with most published studies being conducted in western countries and Asian societies [11]. This study investigated the effect of short-term aerobic exercise on body image and depression among Iranian women. It was hypothesized that aerobic exercise would significantly improve body image attitudes and decrease depression symptoms in comparison with a control group.

## 2. Methods

### 2.1. Participants

Eighty-two participants volunteered to participate in this study. All of them were matched in age, education, and marital status before being assigned to an aerobic exercise group (*n* = 41) or control group (*n* = 41). The subjects were registered in order to begin their program at gymnasiums. The first 41 registrants were selected as exercise group, and the next 41 volunteers were asked to wait for the next four weeks. The study was approved by the Ethics Committee for Human Experiments, University of Welfare and Rehabilitation Sciences, Tehran, Iran, and informed consent was obtained from participants before assignment. Their age ranged from 18 to 45 years, with a mean of 26 years of age (SD = 6.9) in the exercise group and 27 years of age (SD = 6.5) in the control group. Inclusion criteria for participants were as follows:

- aged between 18 and 45 years;
- BDI-II score of 14 or above, [12].

Exclusion criteria were receiving any treatment for depression during the last six months prior to volunteering to participate in the study, participating in any exercise program during the previous three months prior to commencement of the study, and diagnosis of physical and other neurological psychotic disorders.

### 2.2. Measures

Descriptive information of age, education, and marital status were obtained from all participants. Body image was measured by using MBSRQ.

The Multidimensional Body Self-Relation Questionnaire is a self-report inventory for the assessment of self-attitudinal aspects of the body-image construct, which includes The Body Self-Relation Questionnaire (54 items), The Body Areas Satisfaction Scale (BASS) (nine items), The Overweight Preoccupation Scale (four items), and The Self-Classified Weight Scale (two items). The Body Self-Relation Questionnaire consists of three somatic domains: physical appearance, fitness, and health-illness. Within each of these domains, items address either evaluation or orientation to the domain. Participants were asked to indicate their agreement or disagreement with each statement in the survey using a five-point Likert scale. Tests on internal consistency of each subscale yielded a coefficient alpha ranging from 0.73 to 0.95 [13]. Yuen and Hanson (2002) demonstrated that the Multidimensional Body Self-Relation Questionnaire has adequate construct validity [14]. Internal consistency for all subscales in the present sample was acceptable (Cronbach's *α*: 70% to 87%).

BDI-II includes 21-item self-reporting inventory so as to assess the severity of clinical depression and change in depression symptoms across time. This inventory has demonstrated good internal consistency [12] and has been widely used to assess changes in depressive symptoms across time. Cut-off points for scoring the inventory are <14 (minimal), 14 to 19 (mild), 20 to 28 (moderate), and 29 to 63 (severe) depression. The validity and reliability of the Persian version of the Beck Depression Inventory (second edition) have been confirmed [15].

### 2.3. Study Protocol

#### 2.3.1. Exercise Group

The exercise sessions were designed by the exercise instructors and followed the same format with specific intensity and frequency across all sessions. All participants completed both measurements before and after allotted time. Exercise group was then informed about the frame of the sessions and exercise by occupational therapist so that each subject was competent in measuring their wrist pulse.

Resting heart rate was measured several times to ensure subjects had the ability to obtain consistent heart rate values at rest. The resting heart rate measurement was repeated in the next exercise session to further ensure. It was no undue variation in the first and second exercise session measures. Maximum heart rate was estimated from the subject's age as follows: 

Maximum heart rate = 220 – age (in years). 

The overall target of heart rate for the exercise sessions was 70% of maximal heart rate. All participants in the exercise group were required to accomplish their aerobic exercise in front of mirrors to be oriented on details of their movements completely; in addition, the exercise sessions included appropriate music which was adjusted to the intensity of movements which made it possible for all subjects to become well practiced at about 70% of maximal heart rate. 

 Each exercise session included the following sequence of steps.

1. Before starting the exercise, resting heart rate was measured by the participants from the wrist pulse. Aerobic exercise commenced with a “warm up,” followed by dynamic stretching, concentrating on breathing and then continued with slow jogging (10 minutes).
2. In the next phase of the exercise which took 30 to 35 minutes, the movements were performed with higher intensity in the range of 60% to 80% of maximal heart rate. This step included faster unilateral and bilateral hands and feet movements. Some exercise equipments such as bench, box, ball, wooden stick, and weight were used by participants in this regard. Heart rate was measured and recorded after this step (Active heart rate).
3. In the third step, the intensity of movements was decreased, and movements were carried out on mat which included static stretching and muscle relaxation. Immediately after this step, heart rate was measured (15 to 20 minutes).

#### 2.3.2. Control Group

Control group did not participate in any exercises in this duration as waiting list and received the papers which researchers indicated that they would be provided the same exercise protocol in the next four weeks if the results confirmed the exercise effectiveness. It should be noted that all of these participants were newly registered members of gym and had not participate in any other type of exercise.

### 2.4. Analysis

 In this study, SPSS was used to analyse the data. Independent *t*-test was used to compare the effect of aerobic exercise between the exercise group and the control group. Paired *t*-test also was used to compare the heart rate response to aerobic exercise before and after performing each step. Chi-square was conducted to examine the matches of age, education, and marital status among both groups. 

## 3. Results

 According to a Chi-square test, all participants had the same range of education in both groups. Most of them had an undergraduate bachelor degree (*P* = 0.139) (53.3%) and were mostly single (*P* = 0.114) (67.1%), with a mean value of 26.5 SD = 6.9 years in the exercise group and mean value of 26.85, SD = 6.9 years in the control group (*P* = 0.285). 

Maximum target and active heart rate showed that exercise was within the prescribed exercise protocol (see Table 1).  Table 2 depicts the pretest and posttest, mean and standard deviations for each body-image variable (see Table 2). Pretest independent *t*-test confirmed no significant differences between both groups before aerobic exercise in body image subscales while the result of the posttest showed significant differences in appearance evaluation or orientation and health and illness orientation subscales between two groups after undertaking a short-term aerobic exercise program (see Table 3). 

Paired *t*-test conducted on posttest body-image variables revealed significant differences before and after exercise training in the exercise group (see Table 4).  Table 5 depicts the pretest and posttest mean and standard deviations for each body-image variable. Posttest independent *t* test revealed a significant interaction between decreasing depression and exercise training in the exercise group compared with the control group (see Table 5). Paired *t* test conducted on posttest depression variables revealed significant differences between before and after the exercise training program undertaken by the exercise group (see Table 6).

## 4. Discussion

 The exercise group showed a significant decrease in levels of depression coupled with an improved body image in comparison with the control group. The measures of depression in the exercise group had decreased after four-week short-term exercise training program. Symptoms of depression diminished from a moderate level to a mild level in the exercise group. 

 Overall, the ability of aerobic exercise to reduce symptoms of depression in this study is consistent with previous published research [16]. Exercise produces changes in the concentration of several biologically active molecules such as Adrenocorticotrophic hormone, Cortisol, Catecholamines, Opioid peptides, and cytokines [17, 18]. Moreover, some evidence suggests that exercise can modify the concentration of retroactive substances in central nervous system [17, 19]. However, such factors as motivation, expectancy, self-esteem, and human contact may also have influence on the mood of the participants [20–22]. These factors are known to have a direct correlation with the exercise in a social setting [23–25]. 

 It should be noted that some studies do not support the results of the present study regarding reduced depression symptoms with exercise training. Chalder et al. (2012) in a randomized controlled trial concluded that the addition of a facilitated physical activity intervention to usual care does not improve depression outcome or reduce use of antidepressants compared with usual care alone. However, the TREAD (treatment of depression with physical activity) is designed to improve long-term adherence to physical activity [26]. Evidence of a long-term beneficial effect of exercise in patients with clinical depression is limited [27].

 Group activity provided an opportunity for participants to develop more interpersonal relationships with each other and also to reinforce and motivate each other to continue exercise, gain self-efficacy, and improve their mood [21, 28, 29]. All sessions were accompanied by music in order to decrease depression [29]. Another factor that could affect the depression score was the intensity of aerobic exercise [30]. Legrand and Heuze (2007) in their research also confirmed that intense and moderate aerobic exercises appear to have a greater impact on decreasing depression compared with exercise undertaken at a mild intensity [30]. 

 After four weeks, exercise training resulted in significant positive impact on appearance evaluation and orientation and health and illness orientation in comparison with the control group. Improvements in appearance orientation indicated that after exercise training they invested more time on their appearance. Thus, participants in the exercise training program reduced their symptoms of depression and felt well about themselves when they spent more time making sure their appearance was presentable. In addition, higher scores in appearance evaluation after the exercise training program means they had positive feelings about their physical attractiveness. This result revealed that the exercise group became more health conscious and opted to seek medical attention. Several factors can explain this improvement in body-image variables.

 Body esteem is one of the factors that can affect improvement of the body image. The construct of body-esteem places emphasis on person's affective evaluation of their body and is associated with their particular body image [31]. All exercise sessions were undertaken in a gym where mirrors were positioned on the walls and ceiling. Past research has demonstrated that mirror exposure can reduce stress and body dissatisfaction through exercise [32, 33]. Like the depression measures, an exercise program in the form of group activity can also affect the bodyimage, because participants experience more interpersonal relationships and verbal encouragement and persuasion from others [11, 21, 22]. 

 However, scores of other subscales such as fitness orientation or evaluation, health evaluation, and BASS scale did not show any significant differences in the exercise group compared with the control group. Some possible reasons that may explain these results are as follows.

 One factor is the multidimensional nature of physical fitness [2]. The evaluation of physical fitness is characterized by measures of strength, endurance, speed, power output, and flexibility which are governed by a range of body systems including cardiac and respiratory systems. However, the present study emphasized on the heart-rate response and worked on skills and different dimensions of coordination in physical fitness.

A further factor that may influence the results of the present study was the type of exercise undertaken (aerobic exercise) while previous research included resistance exercise for increasing strength and shape of muscles [34]. The duration of exercise is another challenging factor that can affect the result. It seems that for making change in BASS and fitness attitudes, more than four-week exercise is required. It should be noted that this study did not use any weight watcher to avoid interruptions that could affect attitudes of participants especially about BASS scale; furthermore, some studies confirmed that weight perception is more related to depressive symptoms than actual weight [1].

The present research study is limited by a relatively low number of participating subjects, a short exercise training intervention, and the use of a nonclinical participants and nonrandom assignment which may represent a bias. Replication of this study in a larger sample size with random assignment and third group to evaluate regular treatment would be beneficial. Nevertheless, such studies provide a comprehensive insight to the potential value of a short duration exercise program as a treatment strategy. We consider that the present study could be a catalyst to the conduct of larger clinical trials designed to investigate the efficacy of regular moderate exercise in the treatment of depression.

## 5. Conclusion

According to the results of this study, four-week aerobic exercise can effectively reduce depression symptoms and improve some aspects of body-image attitudes in women. Short-term aerobic exercise can be used as an effective method of treatment for these disorders.

## Figures and Tables

**Table 1:** Heart rates of aerobic exercise.

Aerobic exercise scale	Minimum rate	Maximum rate	Mean	SD
MHR	175	202	193.951	6.884
THR (Session 1)	105	121.20	116.370	4.130
THR (Session 2)	148.75	171.70	164.858	5.851
AHR (Session 1)	120	140	118.829	18.33
AHR (Session 2)	120	150	135.365	6.744
RHR (Session 1)	60	90	78.658	8.196
RHR (Session 2)	63	90	79.414	8.285

HR: heart rate; M: maximum; T: target; A: active; R: rest.

**Table 2:** Pretest and posttest, mean and standard deviations of body-image variables.

Multidimensional Body Self-Relation Questionnaire Subscales	Exercise group		Control group	
	Pretest	Posttest	Pretest	Posttest
	Mean ± SD	Mean ± SD	Mean ± SD	Mean ± SD
AE	3.10 ± 0.28	3.53 ± 0.29	3.13 ± 0.46	3.10 ± 0.45
AO	3.41 ± 0.30	3.60 ± 0.26	3.37 ± 0.38	3.33 ± 0.41
FE	3.24 ± 0.50	3.30 ± 0.40	3.13 ± 0.50	3.58 ± 0.58
FO	3.21 ± 0.30	3.30 ± 0.18	3.24 ± 0.33	3.30 ± 0.47
HE	3.18 ± 3.39	3.19 ± 3.35	3.08 ± 0.26	3.19 ± 0.35
HO	3.46 ± 0.46	3.73 ± 0.27	3.45 ± 0.61	3.41 ± 0.60
IO	3.15 ± 0.48	3.54 ± 0.24	3.04 ± 0.42	3.02 ± 0.50
BS	3.34 ± 0.73	3.57 ± 0.66	3.46 ± 0.76	3.48 ± 0.74
OWP	3.06 ± 1.007	2.70 ± 1.78	2.95 ± 1.22	2.99 ± 1.09
SCW	3.42 ± 0.95	2.92 ± 0.96	3.21 ± 0.77	3.20 ± 0.70

AE: appearance evaluation; AO: appearance orientation; FE: fitness evaluation; FO: fitness orientation; HE: health evaluation; HO: health orientation; IO: illness orientation; BS: body areas satisfaction; OWP: overweight preoccupation; SCW: self-classified weight.

**Table 3:** The result of *t*-test among exercise and control group in body-image subscales.

*P value	AE	AO	FE	FO	HE	HO	IO	BS	OWP	SCW
Before exercise	0.715	0.634	0.311	0.688	0.882	0.114	0.289	0.464	0.677	0.285
After exercise	0 < 0.05	0.001	0.141	0.888	0.072	0.003	0.002	0.089	0.167	0.156

*(*P* < 0.05); AE: appearance evaluation; AO: appearance orientation; FE: fitness evaluation; FO: fitness orientation; HE: health evaluation; HO: health orientation; IO: illness orientation; BS: body areas satisfaction; OWP: overweight preoccupation; SCW: self-classified weight.

**Table 4:** The result of paired *t*-test in exercise group in body-image subscales.

*P value	AE	AO	FE	FO	HE	HO	IO	BS	OWP	SCW
Exercise group	0 < 0.05	0 < 0.05	0 < 0.05	0.046	0.040	0 < 0.05	0 < 0.05	0 < 0.05	0 < 0.05	0 < 0.05

*(*P* < 0.05) AE: appearance evaluation; AO: appearance orientation; FE: fitness evaluation; FO: fitness orientation; HE: health evaluation; HO: health orientation; IO: illness orientation; BS: body areas satisfaction; OWP: overweight preoccupation; SCW: self-classified weight.

**Table 5:** Pretest and posttest mean and standard deviations of depression.

	Exercise group		Control group	
	Pretest	Posttest	Pretest	Posttest
	Mean ± SD	Mean ± SD	Mean ± SD	Mean ± SD
Depression	20.58 ± 7.40	5.42 ± 22.04	15.92 ± 5.12	19.95 ± 4.31

**Table 6:** The result of *t*-test among exercise and control group in depression.

*P value	Depression
Before exercise	0.304
After exercise	0.005

*(*P* < 0.05).
